# Posterior Fossa Venous Drainage

**DOI:** 10.3389/fneur.2021.680515

**Published:** 2021-11-08

**Authors:** María Angeles de Miquel

**Affiliations:** Department of Radiology, Interventional Neuroradiology, Bellvitge University Hospital, L'Hospitalet de Llobregat, Barcelona, Spain

**Keywords:** veins, vein of Galen, cerebellum, torcular, petrosal vein, stroke, cerebral vein thrombosis

## Abstract

This paper aims to make simple the evaluation of the main veins related to the brainstem and cerebellum. Posterior fossa venous drainage is best understood in context with its three main collectors: superior: toward the Vein of Galen; posterior: toward the torcular complex; and anterior: toward the superior petrosal sinus. A fourth possible drainage path, often harder to distinguish, is directed toward the inferior petrosal sinus. Veins of these four systems are frequently connected to one another. Despite traditionally being considered less regular than its arterial disposition, posterior fossa venous anatomy follows specific patterns that are easy to identify. The three more representative veins of each venous confluent have been selected, to help in recognizing them angiographically. Since pial large veins are primarily located over the surface of the encephalon, most related anatomical structures can be confidently identified. This is of special interest when angiographic 2D or 3D studies are evaluated and provide fundamental assistance in locating precise structures. To better aid in understanding venous disposition, an overview of embryologic and fetal development is also discussed.

## Introduction

### Embryologic Overview

The brainstem and cerebellum, two main posterior fossa encephalic components, carry a different evolution during the fetal period. For instance, the brainstem already belongs to the neural tube, whereas from one lateral metencephalic plaque at each side, the cerebellum will expand in all directions and converge at the midline. This key distinction influences the subsequent venous disposition.

In general, neural development initially consists of a neural tube of neuroblasts containing a cerebrospinal fluid rich enough to cover the metabolic and energy needs of the surrounding tissue. Originally, cells (neuroblasts) come in contact with both the internal limiting membrane (ventricular or germinal) and the external limiting membrane of the neural tube. The ventricular layer of pluripotential neuroblasts divide rapidly, and subsequently, a ventricular zone, a subventricular zone, and a mantle zone can be differentiated. After a period of mitosis, cells stop dividing and lose contact with the ventricular wall, getting displaced to the mantle zone, where a superficial marginal layer harbors the prolongation with which cells anchor to the external boundary of the neural tube.

**Figure 1:**
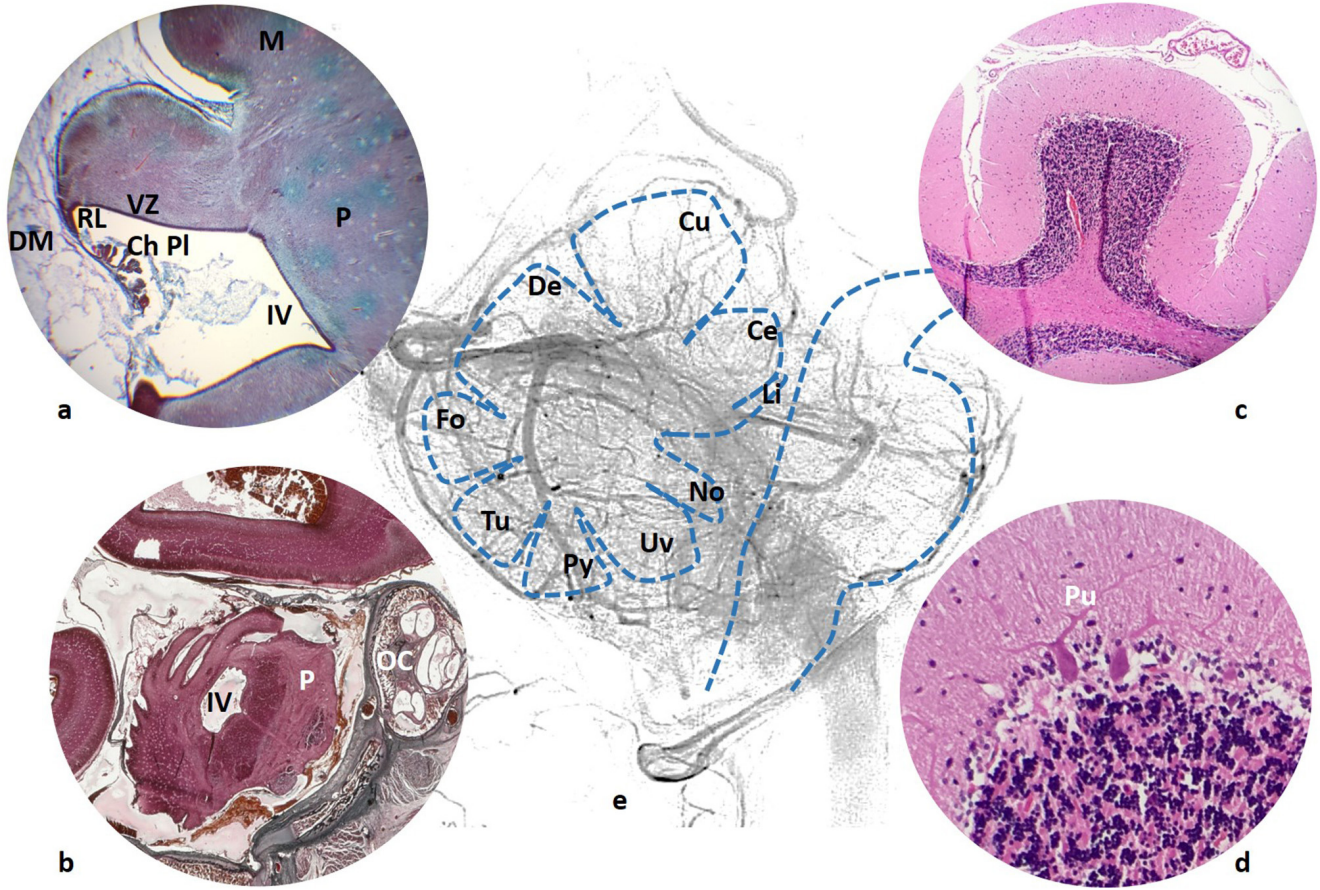
**(a)** 59 mm fetus (10 weeks). Cerebellar ventricular zone (VZ) and rhombic lip (RL), continuous with the choroid plexus (ChPL) of the forth ventricle (IV). Duramater (DM) is still a large tissue surrounding the developing brain. **(b)** 160 mm fetus (20 weeks), cerebellar fissures are clearly seen just posterior to the forth ventricle (IV) and pons (P). A segment of the cochlea is also included in the section (OC). **(c)** Adult cerebellar folia with the typical appearance of the Purkinje cell (Pu) in **(d)**; **(e)** Schema of the vermian lobules (Li, Lingula; Ce, Central; Cu, Culmen; De, Declive; Fo, Folium; Tu, Tuber; Py, Pyramis; Uv, Uvule; No, Nodule) in relationship with the venous network.

All cerebellar neurons are produced at the alar plate of the rhombomere 1 of the rhombencephalon (1). Progenitors in the cerebellar ventricular zone give rise to GABAergic neurons starting with those of the cerebellar nucleus and Purkinje cells (adult Purkinje cells are shown in Figures 1c,d). Cerebellar interneuron progenitors migrate into the developing cerebellar anlage. A secondary germinal zone, the rhombic lip, is established at the junction of the cerebellar ventricular zone and the dorsal roof plate. It gives rise to glutamatergic neurons of the cerebellar nucleus which migrate over the top of the anlage to form the nuclear transition zone, which is a staging zone for cerebellar nuclei assembly. Later, from the rhombic lip (Figure 1a) emerge large numbers of granule neuron progenitors to migrate over the anlage to form the external granular layer, which resides on the pial surface (2).

Our observations of human embryos from three collections (Bellaterra, Blechschmidt and Hinrichsen Collections) confirm what can be seen in the mouse hindbrain development. In the first instance, the cerebrospinal fluid supplies most of the energy needs of developing neuroblasts, while arteries and veins develop and surround the neural tube (3). At the fetal stages, a plexal network of vessels cover the developing encephalic structures (Figure 1b).

At the embryonic and early fetal period, venous drainage is represented by multiple interconnected plexal venous structures located within the dural mesenchyme at the lateral aspect of the future posterior fossa. The young embryo shows the disposition of a large primary head sinus located laterally receiving three main venous stems: anterior plexus and stem (draining the prosencephalon), middle plexus and stem (for the metencephalon), and posterior plexus and stem (for the myelencephalon). The expansion of the craniofacial structures brings the interconnection of the anterior and middle stem components, forming the primitive lateral sinus, which connects to the remnant of the primary head sinus in the sigmoid sinus.

Brainstem veins maintain, more or less, the basic structural pattern of perpendicularly interconnected veins around the neural tissue. Cerebellar drainage, however, follow a particular course of action brought about by the vermian, hemispheric, and ventricular–choroidal tissue expansion.

There is little recent research dedicated to posterior fossa venous development. Old reports of Markowski, Steeter, and Padget (4–6) have been focused on the embryonic period, when the cerebellum is still very undeveloped. Okudera and cols. were interested in the fetal period, but their research in this area was centered aroundthe venous sinuses (7). Research done by the author highlights the important role of the choroid plexus as a directing structure related to further venous arrangement. At early stages, the fourth ventricle is represented by a very large cavity extending laterally and caudally. It is not yet covered by encephalon at its tectal (which will turn into posterior) aspect. With respect to the cerebellar drainage evolution, as Padget stated (7), the pioarachnoidal ventral metencephalic vein is the first to be in relationship with the alar plate of the metencephalon (which will develop into the future cerebellum). The embryonic ventral metencephalic vein becomes the superior petrosal sinus in the adult. This petrosal drainage plays an important role in draining the lateral aspect of the expanded choroid plexus of the fourth ventricle which initially looks like a lateral ribbon extending posterocaudally. Subsequently, the choroid plexus also gets developed at the midline and is drained by veins from the torcular region, foreseeing the development of the inferior vermian veins. The rhombic lip is anatomically connected to the choroid plexus.

It must be emphasized that in most of the embryological and fetal stages, the dura mater does not have the same appearance as in the adult. Instead of a thick fibrous membrane, the developing dura mater consists of large loose connective tissues (Figure 1a) around the neural tube. In embryos between the fifth and the seventh week of gestation only a layer, named “meninx primitiva,” containing most vascular structures, is noted. No distinction can be made between the arachnoid and dural layers. In our specimens, 22 mm (7th week) embryos already show a differentiation between the arachnoid and dura. Vascular venous channels predominantly populate the dura mater, while most arteries are located in the arachnoid tissue. Along the embryologic period, lateral ventricles and the future fourth ventricle are very large cavities.

Most adult pial veins show a final intradural trajectory before entering the sinus, that is, between the dural lining and the corresponding sinus itself. For cerebellar veins draining into the torcula or transverse sinuses through the tentorium, the dural segment tends to be long, to the point that this group of veins of the cerebellar posterior aspect is referred to as the “tentorial sinuses.”

The adult venous cerebellar configuration has been described in 1983 by Duvernoy et al. (8). Two categories of vessels (arterial and venous) were considered, pial and intracortical. About the intracortical veins, they recognized short veins, extremely numerous, related to the molecular layer, of around 10 μm in diameter; medium veins, scarce, originating in the vicinity of the Purkinje layer, and long, more related with the granular layer and white matter; these long veins may show a diameter of up to 90 μm.

On the surface of the cerebellum, they also distinguished between:

- deep pial veins, inside the cerebellar folia, forming venous arches toward larger veins (200 μm) located in the sulci, and
- superficial pial veins: some larger receiving the deep pial vein collectors, and other smaller veins located roughly at the center of the folia.

Some posterior fossa veins are helpful in localizing anatomical structures: the petrosal vein is located at the ponto-cerebellar angle, the precentral vein shows, in the lateral view the anterior margin of the central lobule and culmen, both tonsillar tributaries of the inferior vermian vein turn around the tonsil, sometimes an anterior venous channel shows in the lateral view, the mesencephalon at the interpeduncular cistern, the pons and the medulla (Figure 1a).

The straight sinus, receiving supra- and infratentorial Vein of Galen tributaries, the torcula, and both transverse-sigmoid (lateral) sinuses, represent the main drainage of the posterior fossa. The cavernous sinus, through the superior petrosal sinus and, in some instances, the inferior petrosal sinus, contributes as venous outlets. Additionally, veins of the lateral recess of the fourth ventricle, the restiform body, and anterior medullary veins may also connect with anterior and posterior spinal veins (see **Figure 3**).

**Figure 2:**
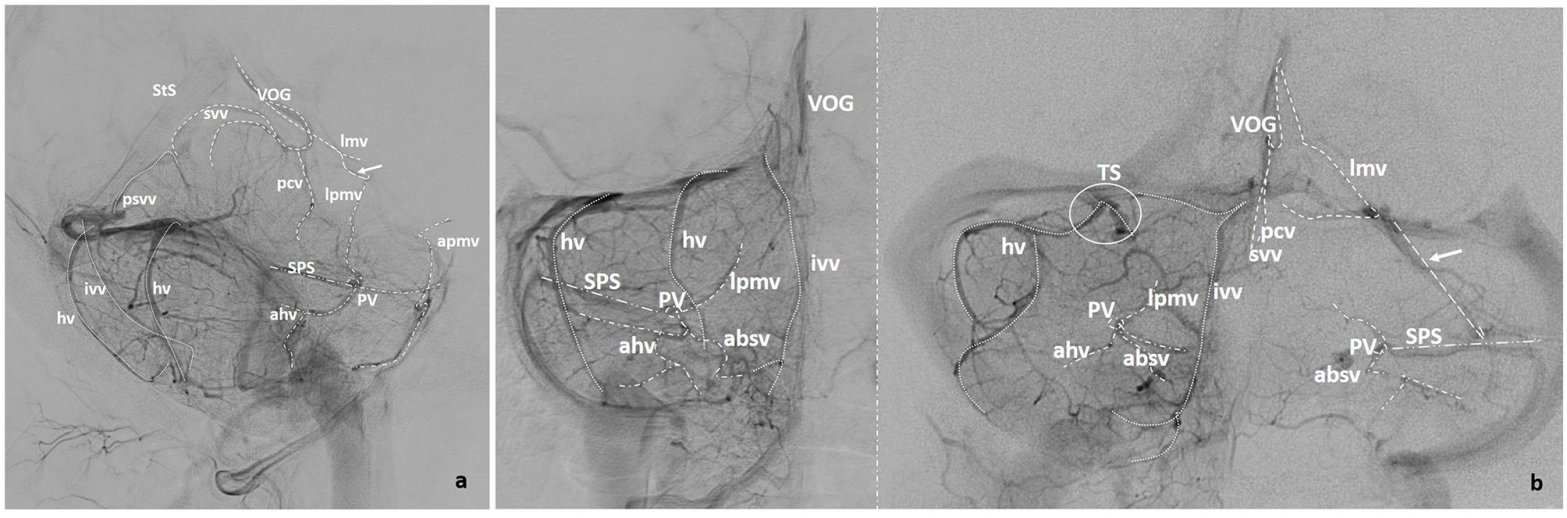
**(a)** Lateral and **(b)** Anteroposterior projections of the veins of the posterior fossa (VOG, Vein of Galen; StS, Straight Sinus; pcv, Precentral Vein; svv, Superior Vermian Vein; lmv, Lateromesencephalic Vein; absv, Anterior Brainstem Veins; lpmv, Lateral Pontomesencephalic Vein; apmv, Anterior Pontomesencephalic Vein; ahv, Anterior Hemispheric Vein; Psvv, Posterosuperior Vermian Vein; hv, Hemispheric Cerebellar Vein(s); ivv, Inferior Vermian Vein; mastv, Mastoid Emissary Vein; PV, Petrosal Vein; SPS, Superior Petrosal Sinus; TS, Tentorial Sinus). Arrows point to bridging veins.

The aim of this review of the venous anatomy of the posterior fossa is not to describe each brainstem or cerebellar vein in detail, but to give a general overview of the main veins normally seen in angiograms (Figures 2a,b) or multiplanar reconstructions of CT angiography (CTA) or MR angiography (MRA). Detailed descriptions of the veins of the brainstem and cerebellum are found elsewhere (9–13).

## Tentorial or Posterior Venous Confluent

This posterior drainage is represented by the cerebellar veins draining toward the torcular region. This also includes cerebellar veins draining to the posterior aspect of the straight sinus and those draining into the lateral sinuses. Pial veins drain through dural venous channels (tentorial sinuses) into the torcula, the lateral sinus, and the straight sinus. As stated by Shapiro et al. (14), these venous channels have been underappreciated in the supratentorial compartment, showing the same appearance as those in the tentorium cerebelli.

Veins of this posterior group include:

- Midline and paramedian veins: the posterosuperior vermian vein, and both inferior vermian veins, normally one on each side, and
- Hemispheric veins.

**Figure 3:**
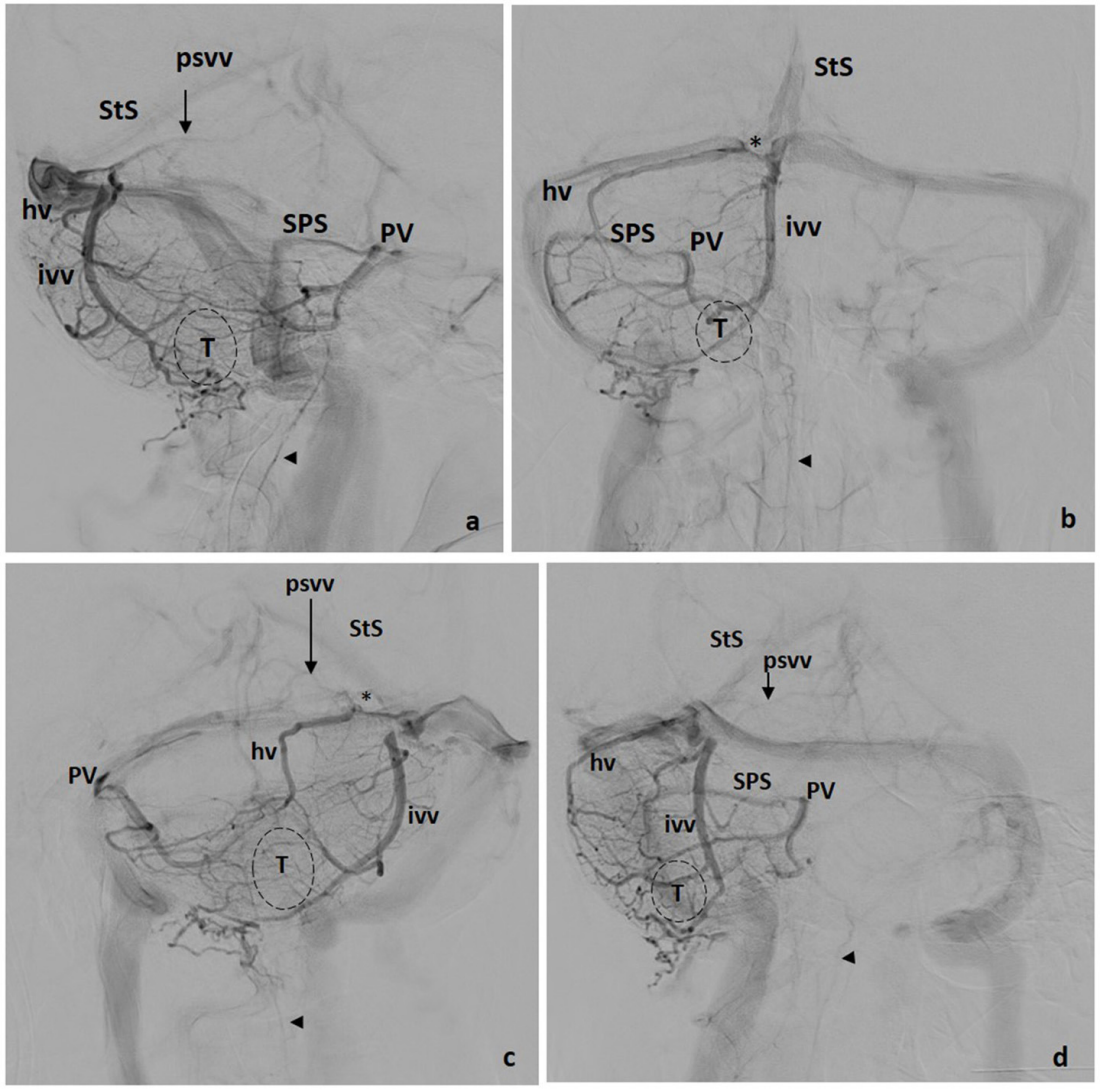
Tentorial drainage. Four views: **(a)** lateral, **(b)** front, **(c)** left oblique, **(d)** right oblique. hv, Hemispheric Vein(s); ivv, Inferior Vermian Vein; psvv, Posterosuperior Vermian Vein; PV, Petrosal Vein; SPS, Superior Petrosal Sinus; StS, Straight Sinus; *, Tentorial Sinus; T, Tonsil; VOG, Vein of Galen; Arrowhead, Anterior Spinal Vein.

A dominant *posterosuperior vermian vein* is infrequent and normally unique at the midline. This vein may be long (draining more than the declive), but usually, it is a short vein joining either the inferior vermian veins (Figure 3a) or draining into the straight sinus or into the torcula itself. In some instances, the posterosuperior vermian vein drains to the vein of Galen, or a superior vermian vein connects the posterior and superior confluent (**Figure 5a**).

**Figure 4:**
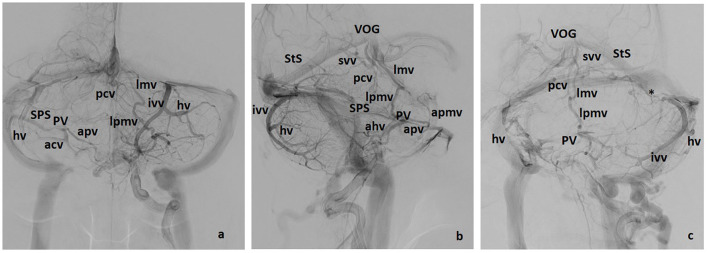
Galenic and Petrosal drainage (including some posterior drainage veins). Three views: **(a)** lateral, **(b)** front, **(c)** left oblique. apmv, Anterior Pontomesencephalic Vein; ahv, Anterior Hemispheric Vein, apv, Anterior Pontine Vein; hv, Hemispheric Vein(s), ivv, Inferior Vermian Vein; lmv, Lateromesencephalic Vein; lpmv, Lateral Pontomesencephalic Vein; pcv, Precentral Vein; psvv, Posterosuperior Vermian Vein; PV, Petrosal Vein; SPS, Superior Petrosal Sinus; StS, Straht Sinus; svv, Superior Vermian Vein; *, Tentorial sinus; T, Tonsil; VOG, Vein of Galen.

*Inferior vermian veins* are usually lengthy, running along the paramedial aspect of the vermis on each side, in or close to the paravermian sulcus. In ~80% of cases their full trajectory is noted in angiographic studies including the para-median PICA territory, draining the corresponding tonsillar region. The junction of a superior and an inferior retrotonsillar vein at what has been named the copular point gives rise to the inferior vermian vein (Figure 3a). The normal configuration is that of an inferior vermian vein either joining an ipsilateral hemispheric vein (Figure 3b) or running independently to drain in the torcular–paratorcular region (Figure 2b). Less frequently, the inferior vermian vein drains in the mid-transverse sinus (Figure 4a) or, exceptionally, in the transverse–sigmoid region. A short vein is uncommon and, in <5% of cases, no inferior vermian vein can be identified. Very uncommonly, the inferior vermian vein crosses the midline to join the torcula or the contralateral inferior vermian vein.

*Hemispheric veins* tend to be more than one in each cerebellar hemisphere, but frequently one is predominant in size. In each hemisphere, posterior draining veins may join in one larger collector with or without connecting to the inferior vermian vein. Drainage of these pial veins through their corresponding tentorial sinuses (Figures 2b, 4a) tend to be directed toward the torcular–paratorcular region in more than 50% of cases, decreasing in frequency to the paramedian third of mid-transverse sinus, to the transverse–sigmoid region and to the straight sinus as points of confluence for those tentorial sinuses.

### Clinical Pearl

The common difficulties that arise when assessing these veins are the following: The inferior vermian vein runs inside the posteroinferior vermian fissure. When seen in the lateral angiographic view, it lies, usually, slightly more anterior than hemispheric veins, but this should be checked in oblique views.

## Superior or Galenic Confluent

**Figure 5:**
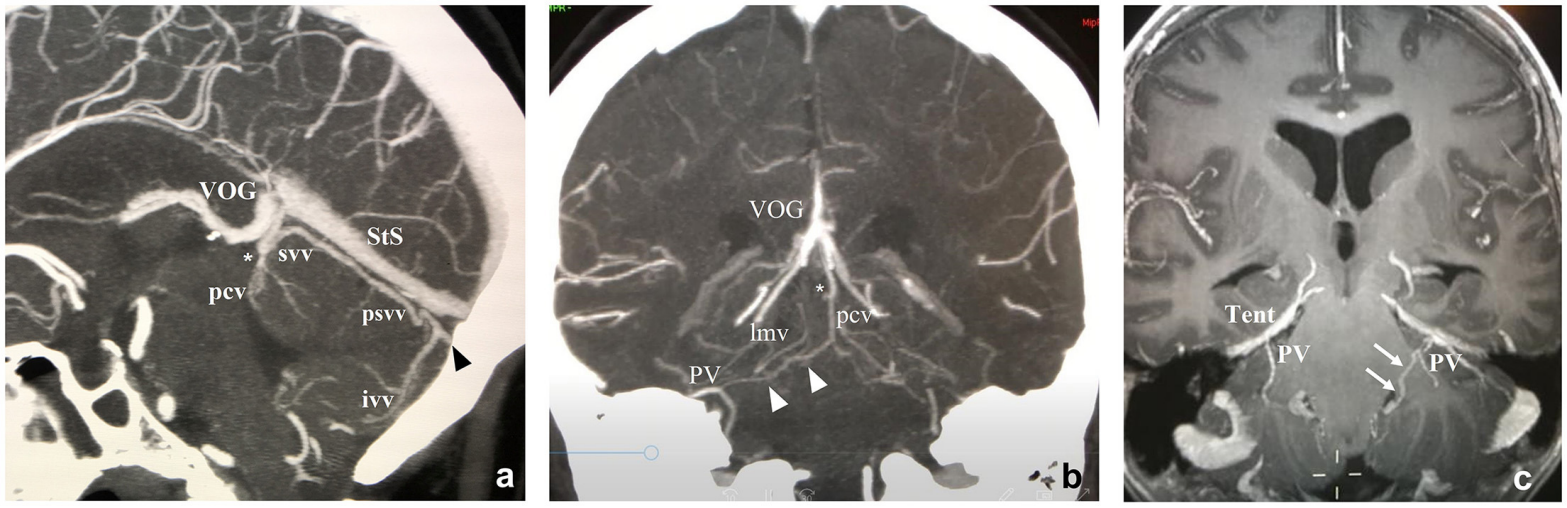
Midline sagittal and retroquadrigeminal coronal CTAngio **(a,b)** and coronal MRAngio **(c)** reconstructions. ivv, Inferior Vermian Vein; lmv, Lateromesencephalic Vein; pcv, Precentral Vein; PV, Petrosal Vein; psvv, Posterosuperior Vermian Vein; StS, Straight Sinus; SPS, Superior Petrosal Sinus; svv, Superior Vermian Vein; Tent, Tentorium; TS, Tentorial Sinus; VOG, Vein of Galen. *Precentral vein and superior vermian vein unite to form a single vein draining into the Vein of Galen. White arrows: Connecting vein between the lateral recess of the IV ventricle and the petrosal vein on the left side. Black arrowhead: Tentorial sinus. White arrowheads: Connecting vein between the petrosal vein and the precentral vein on the right side.

Veins of the brainstem (mainly midbrain–pontine region) and superior aspects of the cerebellum drain to the Vein of Galen and straight sinus representing the superior group. It may be more accurate to use the term “Vein of Galen Complex” since the vein of Galen may adopt different configurations. One of the most common appearances consists of a distinct anteroinferior joining area for the confluence of the internal cerebral vein, the Basal Vein of Rosenthal and/or the Lateromesencephalic vein (Figures 4b, 5a). The junction with the straight sinus is variable, sometimes as a superior branch unrelated to the rest of the joining veins. The vein of Galen may also be a unique venous lake in direct connection to the straight sinus. On the other hand, this Galenic complex may, or may not, show an apparent stenosis at any point before the junction to the straight sinus (Figure 5a).

The main representatives of the superior drainage of the posterior fossa are located at the midline or paramedial venous channels:

- The precentral vein,
- The superior vermian vein, and
- The lateromesencephalic veins and tributaries.

The *precentral vein* is apposed to the anterior aspect of the central lobule of the cerebellum. In ~60% of venograms, it is formed by the union of two paramedial brachial tributaries over the wing and central lobule, one at each side (Figures 4b, 5b), while in some cases (<10%) two independent precentral veins can be found. Its angiographic interest lies in the precentral vein delimits of the anterosuperior margin of the cerebellum, also depicting the collicular region and is almost constant. Huang and Wolf also note that the most caudal, first or fissural, segment of the vein runs behind and parallel to the roof of the upper portion of the fourth ventricle (9).

The *superior vermian vein* is located over the culmen, but its length is variable and may arrive anywhere up to and until the postclival fissure. It tends to be a midline unique vein, but the configuration is also variable, with more than one vein and/or extending mainly over the quadrangular lobe, as a superior hemispheric vein (Figures 2a, 5a).

The most frequent configuration of the drainage of the superior cerebellar region is represented by a superior vermian vein joining the precentral vein (in nearly 80% of our cases) to lead into the vein of Galen complex. Sometimes a collicular vein from the quadrigeminal plate is also recognized.

For the brainstem superior drainage, a long vein turning around the midbrain pontine junction connects with the Vein of Galen Complex. This is referred to as the *lateromesencephalic vein* and runs inside the ambiens cistern (Figures 2a, 4a–c). Huang and Wolf (18) name this vessel the posterior mesencephalic vein, and state that it originates in the interpeduncular fossa or on the lateral aspect of the mesencephalon to join the vein of Galen or the posterior portion of the internal cerebral vein.

In general, veins of the brainstem interconnect in an orthogonal fashion (Figures 1, 2a). Their superior outlet is represented by this posterior or lateromesencephalic vein, which, in some instances, also serves as direct drainage for the basal vein of Rosenthal. In this case, a bridging point is established between supratentorial and infratentorial veins. Since a longitudinal vein frequently runs along the midbrain and the pons, it is more accurate to name it the lateral pontomesencephalic vein (Figures 2a, 3a), which represents a bridging vein connecting the petrosal vein with the lateromesencephalic vein and/or the vein of Rosenthal.

Another interesting vein is the anterior pontomesencephalic vein. It is a longitudinal channel running caudally from the interpeduncular fossa around the anterior aspect of the pons (Figure 4b). It is not constant, but again, when present, delineates, in the lateral angiographic view, the anteromedial aspect of the midbrain, and pons, and may also continue downward toward the medulla. Superiorly, it may connect with the basal vein of Rosenthal.

### Clinical Pearl

The common difficulties that arise when assessing these veins are the following: The precentral vein is not a conspicuous vessel, but in the lateral view should be looked for, since it confidently delineates the anterosuperior margin of the vermis (Figures 1e, 2a,b, 4).

## Anterior or Petrosal Drainage

The petrosal vein draining into the superior petrosal sinus represents a direct derivative of one of the initial pioarachnoidal drainages in the embryo: the ventral metencephalic vein. Being the main venous channel for the lateral pons and cerebellar primordium, it is maintained, essentially, as a constant vein in the adult. Each petrosal vein drains not only the brainstem and the anterior aspect of the cerebellum, but also the neighboring cranial nerves. Venous injection of the posterior fossa shows, at least, one drainage vein for each one of cranial nerves V, VI, VII, and VIII.

In a simplified way, three main venous branches usually converge in the petrous vein. This is a “convenient” landmark, since it helps with roughly indicating the location of the internal auditory meatus and the pontocerebellar angle. These three veins, usually well-recognized in angiograms, meet at the large and short petrosal vein (Figures 2a,b). The petrosal vein is depicted as a “spot” not far from 2 mm in caliber in anteroposterior and lateral views. Then, it joins the superior petrosal sinus, which is a straight dural channel over the edge of the petrous pyramid and tentorium connecting with the transverse-sigmoid angle of the lateral sinus. The anterior connection of the superior petrosal sinus to the cavernous sinus is infrequently seen in angiograms. The main three confluents of the petrosal vein are:

- Lateral pontomesencephalic veins,
- Anterior brainstem veins, and
- Anterior cerebellar veins, superior and inferior.

As noted above, the most frequent lateral vein, running cranio-caudally and longitudinally at the lateral aspect of the mesencephalon and pons, corresponds to the *lateral pontomesencephalic vein* (Figures 2a, 5c). It is an important bridging vein between the basal vein of Rosenthal (when present) or the lateromesencephalic vein, both transversal in direction, and the anterior or petrosal drainage. Additionally, veins coming from the wing of the precentral cerebellar region and superior cerebellar peduncle may join the petrosal vein posterocranially but are rarely well-distinguished in angiograms.

*Anterior brainstem veins* are variable. A transverse directed vein from the anterior aspect of the brainstem to the petrosal vein is usually recognized. The frequent configurations are:

- transverse anterior superior and inferior pontine veins, which run on either side of the emergence of the trigeminal nerve (Figures 2a, 4a,b), or
- a longitudinal venous channel along the midline from the interpeduncular fossa to, sometimes, the anterior veins of the spinal cord, laterally connected perpendicularly with anterolateral veins toward the petrosal vein (Figures 1, 2a), or
- a large anterolateral paramedian vein receiving the orthogonal connecting veins of the midbrain, pons, and medulla (in Figure 4b, these connect to the anterior spinal vein).

The veins of the brainstem also drain those veins of the cranial nerves—most of them toward the pestrosal vein—and are also frequently named based on their location, coursing transversely or longitudinally along the midbrain, pons, and medulla.

*Anterior cerebellar veins* include those of the anterior aspect of the cerebellum that may or may not join in one stem (Figures 2a,b, 4b). The main described veins are the veins of the great horizontal fissure, which may or may not run in this fissure, and the vein of the lateral recess of the fourth ventricle which originates in the region of the dentate nucleus. From the lateral recess of the fourth ventricle, first turns laterally running caudal to the flocculus, and then shows an upward turn toward the petrosal vein. Other veins of the region as the medial tonsillar vein, or the vein of the flocculus are smaller. From a practical point of view, the largest inferior cerebellar vein joining radially those veins of the anterior, middle, and caudal aspect of the cerebellum and its peduncles helps to show the location of the flocculus and cerebellopontomedullary cistern.

### Clinical Pearl

The common difficulties that arise when assessing these veins are the following: The superior petrosal sinus and the petrosal vein are the main anatomical markers of this anterior confluent. The superior petrosal sinus runs over the petrous ridge and its configuration is that of a quite straight venous structure connecting the petrosal vein with the transverse–sigmoid sinus. In consequence, the superior petrosal sinus is angiographically seen as a thin relatively linear venous channel (Figures 2a,b, 3a,b,d, 4a,b). In the frontal view, the location of the superior rim of the petrosal bone depends on the position of the head. In the lateral view, when both cerebellar hemispheres are angiographically depicted, two parallel channels from the petrosal vein to the lateral sinus are easy to find.

## Inferior Petrosal Sinus Drainage

The *vein of the lateral recess of the fourth ventricle* after turning around the cerebellar peduncles, may also continue forward to join the caudal end of the inferior petrosal sinus. This configuration may be difficult to recognize angiographically, but in some instances, such as the dural fistulae can be objectivized.

## Clinical Implications

Arterial ischemic stroke in the posterior fossa would, theoretically, affect infratentorial venous drainage as it does in the supratentorial compartment. Probably, a faster venous drainage of a large ischemic region can be present although underreported. For the venous sector, multiple connecting venous channels frequently prevent venous thrombosis when the occluded vein is secondary. Lesions of main trunks as the petrosal vein, tentorial sinuses, or the vein of Galen complex, however, would easily provoke venous ischemia. A review of isolated vein thrombosis of the posterior fossa presenting as localized cerebellar venous infarctions or hemorrhages found 9 cases among 230 intracranial venous thrombosis. Thrombosis was localized at the straight sinus, lateral sinuses, or the petrosal vein, meaning that in all cases a large venous drainage was affected (15). Anticoagulation remains the main treatment in these cases. Endovascular options as *in situ* venous fibrinolysis or even venous stenting remains, for the moment, as a compulsory treatment option.

On the other hand, postoperative cerebellar swelling (or stroke) may cause severe clinical problems, particularly when the superior petrosal veins are sacrificed during surgery of the posterior fossa (16). Knowledge of the anatomy of the draining patterns of the tentorial sinuses and their draining veins can also benefit the neurosurgeon carrying out repair near or on the cerebellar tentorium (17).

## Final Comments

The present review is based on the radiological approach of posterior fossa drainage. From a neurosurgical approach, Rothon (11) divides the posterior fossa veins into four groups: superficial, deep, brainstem, and bridging veins. The superficial veins are divided on the basis of the three surfaces they drain: (1) tentorial surface by the superior hemispheric and superior vermian vein, (2) the inferior or suboccipital surface is drained by inferior hemispheric and inferior vermian veins, and (3) the petrosal surface is drained by anterior hemispheric veins. The deep veins course in the three fissures between the cerebellum and the brainstem and on the three cerebellar peduncles: veins of the cerebellomesenchephalic, cerebellopontine, and cerebellomedullary fissures, and veins of the superior, middle, and inferior peduncles. In general, veins of the posterior fossa tend to terminate as bridging veins connecting the Galenic, petrosal, and tentorial confluents.

## Author Contributions

The author confirms being the sole contributor of this work and has approved it for publication.

## Conflict of Interest

The author declares that the research was conducted in the absence of any commercial or financial relationships that could be construed as a potential conflict of interest.

## Publisher's Note

All claims expressed in this article are solely those of the authors and do not necessarily represent those of their affiliated organizations, or those of the publisher, the editors and the reviewers. Any product that may be evaluated in this article, or claim that may be made by its manufacturer, is not guaranteed or endorsed by the publisher.

## Abbreviations

Ch Pl: choroid plexus
DM: dura mater
IV: fourth ventricle
M: mesencephalon
OC: otic capsule
P: pons
Pu: Purkinje cell
RL: rhombic lip
Tent: tentorium
VZ: ventricular zone
Li: lingula
Ce: central
Cu: culmen
De: declive
Fo: folium
Tu: tuber
Py: pyramis
Uv: uvule
No: nodule
T: tonsil
Venous structures: Absv: anterior brainstem veins
Ahv: anterior hemispheric vein
Apmv: anterior pontomesencephalic vein
Apv: anterior pontine vein
Hv: hemispheric cerebellar vein(s)
Ivv: inferior vermian vein
Lmv: lateromesencephalic vein
Lpmv: lateral pontomesencephalic vein
Mastv: mastoid emissary vein
Pcv: precentral vein
Psvv: posterosuperior vermian vein
PV: petrosal vein
SPS: superior petrosal sinus
StS: straight sinus
Svv: superior vermian vein
TS: tentorial sinus
VOG: Vein of Galen.
